
JOURNAL INFORMATION
==============================
NLM Title Abbreviation: ScientificWorldJournal
Journal ID: ScientificWorldJournal
NLM Journal ID: 101131163
Journal ID: TSWJ

The Scientific World Journal

ISSN: 2356-6140
EISSN: 1537-744X
Publisher: Wiley

ARTICLE INFORMATION
==============================
PMCID: PMC6084740
PMID: 30147477
DOI: 10.1100/tsw.2001.161
Article ID: 931927
Article version: 1
Subjects: Short Report

Heat Shock Protein 70 Protects from Caspase-Independent Programmed Cell Death via Suppression of Stress Kinase JNK

Gabai Vladimir L. 1 *
Meriin Anatoli B. 1
Yaglom Julia A. 1
Mosser Dick D. 2
Sherman Michael Y. 1
1 Boston Biomedical Research Institute, 64 Grove Street, Watertown, MA 02472, USA
2 Biotechnology Research Institute, Montreal, Quebec H4P 2R2, Canada
*Vladimir L. Gabai: gabai@bbri.org
Publication date: 2001
Electronic publication date: 2001 Oct 12
Volume: 1
First page: 36
Last page: 36
Copyright: Copyright © 2001 Vladimir L. Gabai et al.
Copyright year: 2001
License: This is an open access article distributed under the Creative Commons Attribution License, which permits unrestricted use, distribution, and reproduction in any medium, provided the original work is properly cited.
License URL: https://creativecommons.org/licenses/by/3.0/

==============================
Miami *Nature Biotechnology* Short Reports
*TheScientificWorld* (2001) 1(S3), 36SR
ISSN 1532-2246; DOI 10.1100/tsw.2001.161

### HEAT SHOCK PROTEIN 70 PROTECTS FROM CASPASE-INDEPENDENT PROGRAMMED CELL DEATH VIA SUPPRESSION OF STRESS KINASE JNK

Vladimir L. Gabai[*,1], Anatoli B. Meriin[1], Julia A. Yaglom[1], Dick D. Mosser[2], and Michael Y. Sherman[1]

[1]Boston Biomedical Research Institute, 64 Grove Street, Watertown, MA 02472, USA;
[2]Biotechnology Research Institute, Montreal, Quebec H4P 2R2, Canada
* gabai@bbri.org

**INTRODUCTION.** A major inducible heat shock protein Hsp70 (Hsp72) is known to protect cells from diverse apoptosis-inducing stimuli but the mechanism of the protection has not been established yet (1,2). We have previously found that Hsp70 may inhibit apoptotic signal transduction at an early step, via suppression of stress kinase JNK (3); in addition, recent data indicate that Hsp70 may block directly apoptosome formation and caspase-9/3 activation (4,5). However, besides "classical" caspase-dependent apoptosis, there is a programmed cell death that is caspase-independent. Here we studied whether Hsp70 can protect from such type of cell death and what is the mechanism of the protection.

**METHODS.** To increase levels of Hsp70 we used adenovirus expressing human Hsp72 under control of tet-promoter, and adenovirus expressing dominant-negative mutant of SEK1 was used to inhibit JNK activation. Cell death was assessed by acridine orange/ethidium bromide staining under fluorescent microscope, or by poly-ADP ribose (PARP) cleavage and caspase-3 activation by immunoblotting. Activities of MAP kinases (ERK1/2, p38, and JNK) were measured by immunoblotting with antibodies to phosphorylated (activated) form of the kinases.

**RESULTS.** Although the protective effect of Hsp70 against caspase-dependent apoptosis is well established, little is known about its role in caspase-independent cell death. We studied the effect of Hsp70 in two models of caspase-independent cell death: heat-induced killing of normal human fibroblasts and death of myogenic cells after transient ATP depletion. Morphology of heat-treated IMR90 diploid fibroblasts resembled that of apoptotic cells (e.g., after TNF or staurosporine treatment), and the plasma membrane remained intact. Morphology of H9c2 myogenic cells after transient ATP depletion was also similar to that of apoptotic cells, but some cells lost plasma membrane integrity. Both forms of cell death were not accompanied either by caspase-3 activation or PARP cleavage. Moreover, pancaspase inhibitor zVAD-fmk did not protect either from heat-induced killing of fibroblasts or ischemic death of H9c2 cells. Both heating and transient energy deprivation activated several MAP kinases, Erk1/2, p38, and JNK in these cells. As with classical apoptosis, inhibition of "survival" kinase, Erk1/2, decreased cell survival in both models. In contrast, inhibition of JNK (but not p38) almost completely protected from heat-induced killing of fibroblasts and markedly reduced death of H9c2 after energy deprivation. These types of caspase-independent cell death were effectively prevented by increased levels of Hsp70. Hsp70 overexpression greatly diminished activation of JNK after heat shock or transient energy deprivation; on the other hand, it did not increase activity of Erk1/2. Therefore, the protection of cells from the caspaseindependent death appears to be due to the suppression of JNK, and it is unrelated to the regulation of survival kinase Erk1/2.

Studying possible mechanisms of Hsp70-mediated JNK inhibition, we found that Hsp70 overexpression did not suppress activity of upstream components of JNK signaling cascade (e.g., SEK1). However, Hsp70 markedly alleviated inhibition of JNK dephosphorylation (i.e., deactivation) caused by heat shock or transient ATP depletion.

**CONCLUSION.** The data obtained strongly suggest that Hsp70-mediated suppression of JNK stress kinase is implicated in Hsp70-mediated protection from caspase-independent cell death of fibroblasts under heat shock and myogenic cells under transient energy deprivation. Our results also indicate that the cardioprotective effect of Hsp70 may be associated with suppression of JNK.

**REFERENCES.**

1. Gabai, V.L., Meriin, A.B., Yaglom, J.A., Volloch, V.Z., and Sherman, M.Y. (1998) FEBS Lett. 438, 1-4
2. Jaattela, M. (1999) Exp. Cell Res. 248, 30-43
3. Gabai, V.L., Meriin, A.B., Mosser, D.D., Caron, A.W., Rits, S., Shifrin, V.I., and Sherman, M.Y. (1997) J. Biol. Chem. 272, 18033-18037
4. Saleh, A., Srinivasula, S.M., Balkir, L., Robbins, P.D., and Alnemri, E.S. (2000) Nat. Cell Biol. 2, 476-483
5. Beere, H.M., Wolf, B.B., Cain, K., Mosser, D.D., Mahboubi, A., Kuwana, T., Tailor, P., Morimoto, R.I., Cohen, G.M., and Green, D.R. (2000) Nat. Cell Biol. 2, 469-475
